# Enhancing the learning of sports skills through verbal feedback

**DOI:** 10.3389/fspor.2025.1519365

**Published:** 2025-04-08

**Authors:** Tomasz Niźnikowski, Paweł Arnista, Jerzy Sadowski, Andrzej Mastalerz, Oscar Romero-Ramos, Emilio Fernández-Rodríguez, Weronika Łuba-Arnista, Michał Biegajło, Paweł Różański, Ewelina Niźnikowska, Andrzej Karaś, Piotr Kuśmierczyk, Marta Nogal

**Affiliations:** ^1^ Department of Gymnastics, Faculty of Physical Education and Health, Józef Piłsudski University of Physical Education in Warsaw, Biała Podlaska, Poland; ^2^ Faculty of Health Sciences, Lomza State University of Applied Sciences, Łomża, Poland; ^3^ Faculty of Physical Education, Józef Piłsudski University of Physical Education in Warsaw, Warsaw, Poland; ^4^ Sport Department, Faculty of Education Sciences, University of Malaga, Málaga, Spain; ^5^ Department of Tourism and Recreation, John Paul II University in Biała Podlaska, Biała Podlaska, Poland

**Keywords:** female gymnasts, verbal feedback, key elements, round-off back somersault, artistic gymnastics

## Abstract

**Introduction:**

A critical aspect of motor skill acquisition is the feedback provided to the learner. Numerous studies have attempted to identify the most effective approach to providing feedback to individuals in the process of learning or refining motor skills. We investigated to determine the effectiveness of verbal feedback in learning the round-off back somersault on the balance beam with stable landing.

**Methods:**

The research material consisted of female gymnasts (*n* = 16). They were randomly assigned to one of two groups: FKE group (*n* = 8) with feedback on key elements, or FAE group (*n* = 8) with 100% feedback on all errors made in the phase structure of the task.

**Results:**

Based on research, it was established that key elements could be identified in the preparatory, main and final phases. Mixed ANOVA showed that significant differences (*p* < 0.05) were noted due to the teaching method used (Group x Time factor) when performing the gymnastic routine of round-off back somersault with stable landing on the balance beam. Based on the Welch t test for independent data it was revealed that the group with feedback on the key elements obtained significantly higher mean scores from judges at the end of the experiment.

**Discussion:**

Coaches should strategically provide feedback on key errors to optimize training and potentially improve competition performance. The study concludes that the effectiveness of learning the round-off back somersault on the beam is enhanced by purposeful verbal feedback. Reducing the frequency of feedback and focusing on key elements rather than addressing all errors proves more beneficial. Further research is needed to study the role of feedback directed at key elements in learning complex routine with multiple degrees of freedom among elite athletes in practical settings.

## 1 Introduction

One of the main issues that coaches have to deal with is how to facilitate the process of learning motor skills and enhance the effectiveness of training processes. Feedback (its provision, reception and processing) plays a significant role in this context. Numerous studies show that the use of different types of feedback may either improve or impair the process of learning motor skills (1–6). The current state of knowledge in this area indicates that when performing motor tasks, learners use intrinsic feedback. This type of information can be augmented with extrinsic feedback, which may lead to an improvement in motor skill performance. To support this thesis, some studies have been published that focused on motor skills and the use of different types of feedback such as bandwidth feedback in a gymnastic skill (7), novel upper limb joint coordination pattern with augmented auditory feedback (8), effects of feedback after good and poor trials in continuous motor tasks (9), different feedback frequencies in learning an arm movement sequence (10), effects of verbal, haptic, and combined (verbal and haptic) feedback when learning a novel gymnastic parallel bars task (11), feedback modalities in the motor learning of complex tasks (12).

Sports practitioners and theorists claim that verbal cues are an important part of a training process. Verbal feedback provided properly may produce beneficial effects in training and, first and foremost, in competitions. Extrinsic feedback constitutes a supplementation of intrinsic feedback of a learner (13). Coaches may provide their athletes with knowledge of result (KR) and knowledge of performance (KP). It is believed that KP gives learners more accurate information on kinematics and movement pattern; therefore, coaches use this type of feedback more often in a training process (6).

The volume of feedback during its delivery was a necessary variable (14). It is believed that extrinsic feedback is effective since it provides the learner with adequate response, reduces errors and maintains goal orientation. Constant feedback may facilitate constant control through creating direct connections between the desired outcome and intrinsic feedback. However, even though constant feedback contributes considerably to an increase in motor skill learning effectiveness, when it is not provided in retention and transfer tests, learning effectiveness decreases markedly. These findings have strong relevance for training and simulation where retention and transfer performance is the ultimate goal (14). Constant feedback may discourage learners from using intrinsic feedback (15, 16). Reduced frequency of feedback enhances motor skill learning because trials without feedback may cause the learner to engage additional significant cognitive processes such as those associated with error detection (17). However, other studies using relatively complex tasks yielded mixed results. For example, in contrast to simple laboratory tasks used in many studies, Wulf and Shea (18) found that reducing relative feedback frequency did not enhance learning of the ski simulator task. In fact, providing concurrent feedback on 100% of the practice trials turned out to be most effective for learning.

In one study on verbal feedback, reduced frequency of feedback was applied so that the Focus was on the so-called key elements of movement, which led to an improvement in the effectiveness of complex motor skill learning (19). Key elements of movement are the most important and characteristic body postures that are the main determinants of motor skill performance (20). It is implied that such verbal cues on key elements of motor skill performance produce positive learning outcomes in athletes who can notice and understand their errors. However, opinions vary as to an optimal amount and purposefulness of feedback in the process of learning difficult motor skills (6, 8–10, 21, 22). There is a scarcity of data on providing verbal feedback on key elements of those gymnastic skills which correlate with experts’ ratings.

The aim of the study was to determine the effectiveness of verbal feedback in learning the round-off back somersault on the balance beam with stable landing.

Given the previous research, we hypothesized that the most effective feedback in learning motor learning the round-off back somersault on the balance beam with stable landing would be verbal cues on key elements of motor skill.

## 2 Materials and methods

### 2.1 Participants

Sixteen skilled male and female junior gymnasts participated in the study. Height was 152.8 cm (± 3.92 cm), body mass 44.5 kg (± 4.24 kg) and age 14.1 years (± 0.6years), respectively. The participants were randomly assigned in equal numbers into two groups; an experimental group (FKE), who received feedback on errors made in key elements of sports technique, and a control group (FAE), who received feedback on all errors committed in the motor skill structure.

The study was conducted following the Declaration of Helsinki, and approved by the Senate Scientific Research Ethics Committee of Józef Piłsudski University (protocol code: SKE 01-11/2018). All the gymnasts were informed in a written and oral manner about the tests that would be carried out in the study.

Throughout the experiment, the participants learnt and improved particular skills connected with the performance of the round-off back somersault on the balance beam with stable landing (RBSBB). These skills were part of routines performed during competitions.

Duration of experiment was 6 weeks, with five 120-minute training sessions per week on Monday, Tuesday, Wednesday, Friday and Saturday. After a standard warm-up, each participant performed 3 sets × 3 repetitions of the routine during each practice session. Feedback was provided directly after each set of 3 repetitions (Table 1). The conducting the experiments were the coaches of the National Team of the Polish Gymnastics Association.

**Table 1:** Analysis of correlations between measurements performed during the round-off back somersault on the balance beam with stable landing and judges’ ratings.

Joint angles of resultant velocities	Pre-test		Post-test		Retention test	
	rho	*p*	rho	*p*	rho	*p*
Shank-thigh angles LBP	0.3	0.265	**0** **.** **67**	**0**.**005**	−0.06	0.84
Thigh-trunk angles LBP	−0.26	0.335	0.09	0.743	**−0**.**63**	**0**.**009**
Trunk-arm angles MBP	**0**.**44**	**0**.**09**	−0.44	0.089	0.11	0.697
Thigh-trunk angles LBP	−0.2	0.466	**0**.**63**	**0**.**01**	**−0**.**61**	**0**.**012**
CG LBP	**0**.**54**	**0**.**032**	0.26	0.339	−0.33	0.206
Elbow joint velocities MBP	−0.14	0.606	0.32	0.226	**−0**.**5**	**0**.**051**
ankle joint velocities LBP	−0.03	0.904	−0.06	0.814	**0**.**63**	**0**.**008**

Significant changes are shown in bold at *p* < 0.05, rho—correlation coefficient.

Participants received KP of each repetition in a given set. There was a 5-minute interval between the sets. In total, each participant performed 270 repetitions.

Technical skills of the gymnasts were tested at the beginning of the experiment (pre-test) and immediately after (post-test), while one week later training outcomes were evaluated. The concordance coefficient was used to estimate the agreement between experts’ ratings (r = 0.848).

To assess sports technique, APAS 2000 modular analyser was employed. The analyser makes it possible to register, measure and analyse 2D movement owing to the use of two professional video recorders (240 frames/s). The measurement error of this equipment is 2%–3%.

**Figure 1:**
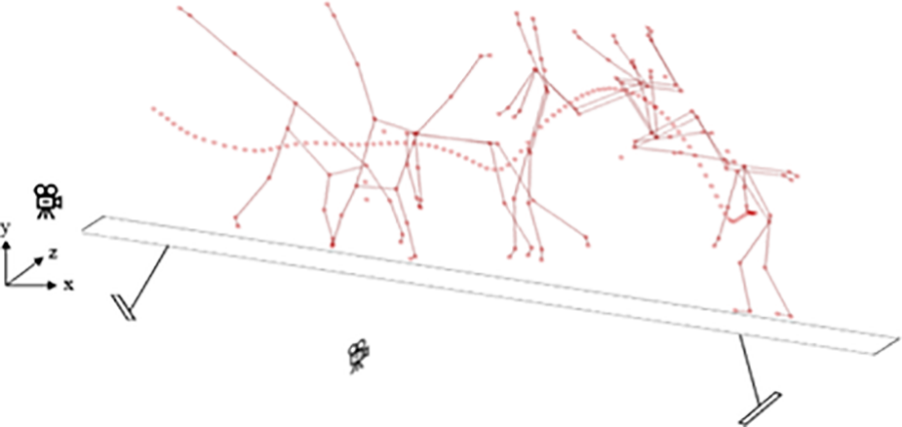
The round-off back somersault on the balance beam with stable landing and camera positioning.

The round-off back somersault on the balance beam with stable landing is illustrated in Figure 1.

Based on the registered gymnastic routines, the following were calculated: Parameters of trajectories of body biolinks movements and the centre of gravity (CG); Body positioning in rotary movements; Time characteristics of movement phases, exercise duration; Velocities and joint angles. To obtain extra information about key elements of sports technique, a biomechanical analysis was carried out. A detailed analysis of exercises and their dynamic combinations were performed.

Statistical analyses were conducted using R 3.6.2. A 2 × 3 mixed ANOVA was applied due to the quantitative nature of the variables and minimal skewness. This included feedback type (FKE vs. FAE) as an interpersonal factor and test timing (pre-test, post-test, retention test) as an intrapersonal factor. The ANOVA assessed main effects of feedback and testing, and their interaction. Significant main effects suggest differences in mean scores across groups or tests, while significant interactions indicate the feedback type's impact on training effectiveness.

*post hoc* tests, without correction due to the small sample size, were used to compare means. Student's *t*-tests for dependent data assessed intra-group differences, while Welch's *t*-tests for independent data evaluated inter-group differences for each test. Spearman's rho was used to examine correlations between judges’ ratings and motor skill metrics. The significance level was set at *p* < 0.05.

## 3 Results

### 3.1 Identifying key technical elements of the skill

In the first stage, place, the study focused on identifying key technical elements of the skill under study. Three key elements were identified in the phase structure of the motor skill understood as the most characteristic and important body postures that determine the motor skill performance.

**Figure 2:**
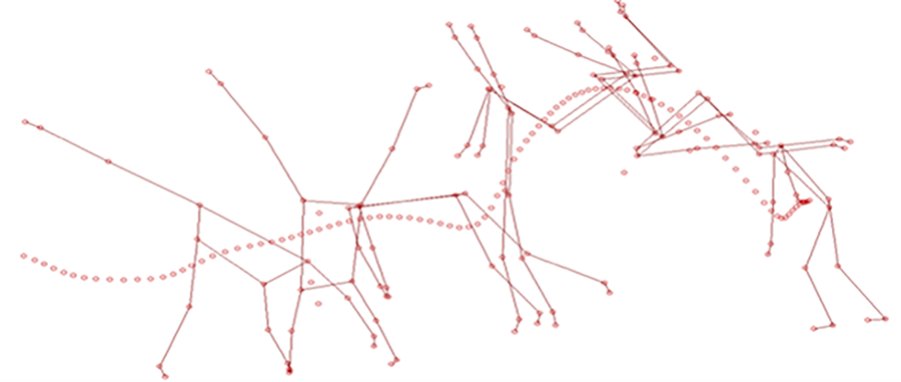
Kinematic structure and COM trajectories in a female gymnast while performing the gymnastic routine of round-off back somersault with stable landing on the balance beam.

Figure 2 shows a kinematic structure of trajectories of COM displacements in a female gymnast while performing the gymnastic routine of round-off back somersault with stable landing on the balance beam.

In the preparatory phase, biomechanical parameters characterising the launching body posture (LBP). It was noted that in the final phase of the round-off, before the back somersault with stable landing on the balance beam (0.08 s), LBP is manifested by a bent body posture (hip-trunk angle of 171.67°). LBP was performed before a vertical body position was reached, on toes, in tandem stance (feet positioned one behind the other), with arms upward and forward. In the main phase of the structure of the motor skill, multiplication of body postures (MBP) was identified (0.28 s). The thigh-trunk angle was 100.05°. In the final phase (0.62 s), landing body posture was distinguished (LBP), during which gymnasts prepared for landing. Stable landing (1.04 s) was characterised by the thigh-trunk angle of 130.08°. It was noted that gymnasts demonstrated proper elasto-rigid feet interaction with the beam, which helps to assume optimal LBP in the half-squat position, with bent trunk and arms forward, downward and outward.

Identifying whether key elements of the phase structure of the round-off back somersault on the balance beam with stable landing correlated with judges’ ratings.

Table 1 shows only significant Spearman's rho correlation coefficients and their respective levels of significance for correlations between measurements performed during the round-off back somersault on the balance beam with stable landing and judges’ ratings, separately for each stage of the study.

There were positive significant and moderately strong correlations between judges’ ratings at pre-test and the trunk-arm angle in the multiplication of body postures and the distribution of the centre of gravity displacements. At post-test, positive significant and moderately strong correlations were noted between the shank-thigh angle in the launching body posture and the thigh-trunk angle UCL. At retention, negative significant and moderately strong correlations were found between judges’ ratings and the thigh-trunk angle in the launching and landing body postures as well as resultant velocities of elbow joints in the multiplication of body postures. Moreover, positive significant and moderately strong correlations were observed between judges’ ratings and resultant velocities of ankle joints in the landing body posture.

Experts’ ratings for the performance of the round-off back somersault on the balance beam with stable landing

**Figure 3:**
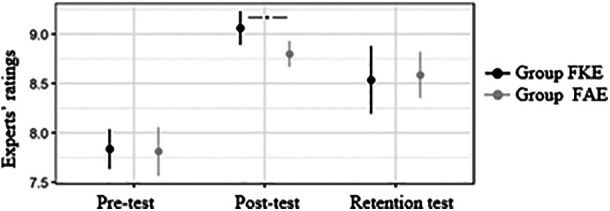
Experts’ ratings for the performance of the round-off back somersault on the balance beam with stable landing.

Learning outcomes at retention for the round-off back somersault on the balance beam with stable landing are shown in Figure 3.

The Welch *t*-test for independent data collected at pre-test revealed not significant differences between the groups [t(13,4) = 0.22; *p* > 0.05]. Based on mixed ANOVA, significant Test Time effect was noted [F(2,28) = 82.92; *p* < 0.001; eta^2^ = 0.82]. No Group effect [F(1,14) = 2.03; *p* > 0.05; eta^2^ = 0.03] as well as no Group × Test Time interactions were found [F(2,28) = 1.74; *p* > 0.05; eta^2^ = 0.09]. Based on the Welch *t*-test for independent data, it was shown that at post-test, FKE group (with feedback on key elements) received significantly higher mean scores from judges than FAE group (with feedback on all errors during motor skill performance) [t(13,19) = 3.48; *p* < 0.01]. At retention, differences were not significant [t(12,42) = −0.34; *p* > 0.05].

## 4 Discussion

The aim of the study was to determine the effectiveness of verbal feedback in learning the round-off back somersault on the balance beam with stable landing. The current study current study focused on identifying key elements of sports technique in complex motor skills and providing feedback on those elements. It was found that verbal feedback on errors in key elements helps to increase the effectiveness of learning and improving complex motor skills in gymnastics (7).

In recent years, artistic gymnastics has seen an increase in the level of difficulty of exercises as well as gymnastic routines during competitions. Therefore, training solutions that aim at improving processes of learning motor skills are of particular importance. Coaches stress the need to apply highly effective, long-lasting and flexible methods of teaching motor skills. The impact of verbal feedback is one of the factors influencing the effectiveness of methods in training processes (23).

By providing verbal feedback on key elements, we influenced the effectiveness of learning the round-off back somersault on the balance beam with stable landing. It was confirmed by significant differences revealed through the method of teaching RBSBB. It was noted that the group with feedback on key elements received significantly higher mean scores from judges at post-test and retention test. Similarly, Lai and Shea (24) claimed that reduced frequency of knowledge of results (50%) produced better learning outcomes than full (100%) feedback. Their findings are in line with the results obtained by Niźnikowski and Sadowski (25), who studied the effects of verbal feedback on the learning dismount double salto backward piked from uneven bars by highly-skilled female gymnasts. They found significant differences (*p* < 0.001) between mean scores of each group, which may indicate that increased frequency of verbal feedback on technical errors exerts a negative influence on the process of learning motor skills. Reducing feedback to key elements only ensures considerably higher learning outcomes. Athletes, particularly during competitions, can process a limited amount of information only; therefore, both the quantity and quality of feedback should be reduced to direct instructions related to key elements in sports technique of gymnastic routines. Furthermore, feedback on errors may motivate an athlete to increase their effort to improve a given motor skill. It was confirmed by Ede et al. (26), who analysed a motivating factor on effort in an endurance task.

Providing feedback on errors committed during the performance of the round-off—double salto backward tucked during beam dismount produced better learning outcomes compared feedback on all technical errors (27). It should be noted that their study included elite female gymnasts. The application of this method resulted in gymnasts being aware of their own technical value and achieving remarkable results in competitions (28). Thus, it may be assumed that technical training of skills performed exercises is more effective if it is based on a biomechanical analysis of key elements which form the basis for developing teaching programmes (20, 29). It seems that reducing feedback to errors in key elements only is more effective in learning and improving gymnastic skills than feedback on all errors. Verbal feedback exerts a considerable influence on the process of learning and contributes to proper task performance. Verbal feedback provides important information that supports motor skill learning (30). Knowledge of performance and key elements is a determinant of sports results and it contributes to the optimisation of a training process (27). Verbal feedback is perceived as the main method of improving performance, boosting athletes’ self- confidence and monitoring progress in teaching complex motor skills (24, 25).

One of the ideas to optimise technical training in gymnastics is to enhance the effectiveness of teaching and learning motor skills through reduced frequency of feedback on errors committed in key technical elements. It seems that further research on technical preparation of female gymnasts is needed.

The findings of the present study point to a possibility of offering coaches teaching methods that increase the effectiveness of learning complex motor skills and indicate a possibility of applying such solutions in groups of female gymnasts both in training and during competitions.

Further research is needed to study the role of feedback directed at key elements in learning complex routine with multiple degrees of freedom among elite athletes in practical settings. It remains to be investigated to what extent the principles for learning a specific complex motor routine can be generalised to learning other complex motor routine.

## 5 Conclusions

The study concludes that the effectiveness of learning the round-off back somersault on the beam is enhanced by purposeful verbal feedback. Reducing the frequency of feedback and focusing on key elements rather than addressing all errors proves more beneficial. Coaches should strategically provide feedback on key errors to optimize training, minimize injuries, and potentially improve competition performance.

## Data Availability

The raw data supporting the conclusions of this article will be made available by the authors, without undue reservation.
